# Dietary risk of donated food at an Australian food bank: an audit protocol

**DOI:** 10.1186/s40795-023-00719-8

**Published:** 2023-06-05

**Authors:** Sharonna Mossenson, Roslyn Giglia, Claire E. Pulker, Miranda Chester, Christina M. Pollard

**Affiliations:** 1 School of Population Health, Curtin University, Bentley, WA 6102 Australia; 2 Foodbank of Western Australia, Perth Airport, WA 6105 Australia; 3 East Metropolitan Health Service, Perth, WA 6000 Australia; 4 Enable Institute, Curtin University, Bentley, WA 6102 Australia

**Keywords:** Food assistance, Food relief, Food bank, Donation, Dietary risk, Nutrition, Food safety

## Abstract

**Background:**

Sufficient, safe and nutritious food is unattainable for many people experiencing severe food insecurity, putting them at dietary risk. Food banks, a growing part of the charitable food system (CFS), are the main source of food relief in developed countries. Donations of surplus, unsalable food from supermarkets, producers and manufacturers is the main source of the food supply, and this can be unpredictable, insufficient and inappropriate. The universal performance indicator of food-banking success is a weight-based measure, complemented by various initiatives to track the nutritional quality of food provided. There is currently no method that assesses the dietary risk of donated food related to nutrition and food safety. This protocol describes a method developed to identify and assess the dietary risk of donated food at an Australian food bank including the type, amount, nutrition quality, and food safety.

**Methods:**

An audit of all food donated to a food bank servicing one Australian state was conducted over five consecutive days in May 2022. The audit process used a mobile device to take photographs of all incoming deliveries to the food bank. The images were manually annotated to document the type of food, product information (brand and product name, variety), the donor’s name, weight (kilograms), and date-marking details. Data was extracted from the photographs and assessed against pre-determined dietary risk criterion for food safety (date marking, damaged packaging, visible food spoilage) and nutrition quality according to the principles of the Australian Guide to Healthy Eating, and the NOVA classification of level of processing.

**Discussion:**

Fifteen hundred images were required to assess the dietary risk of 86,050 kg of donated food. There were 72 separate donations, largely from supermarkets and food manufacturers. Data analysis will enable identification of dietary risk, particularly for nutrition quality and food safety. This is important given the absence of food regulation for CFS donations, and the vulnerability of the client group. This protocol highlights the need for more transparency and accountability from food donors, about the food they donate.

## Background

Emergency food relief provided by the charitable food system (CFS) [1] aims to address the immediate needs of people experiencing food insecurity [2]. Defined as the limited or uncertain physical, social or economic access to sufficient, safe, nutritious and culturally appropriate food [3], food insecurity is an important, and increasing public health issue [4]. The CFS comprises a diverse network that varies across countries and incorporates numerous organisations, operations and food service models [5]. Food banks are considered the most prevalent form of charitable food provision [6]. Food banks are 'indirect services’; the organisations responsible for sourcing, banking and distributing surplus food to ‘direct services’ [7], community organisations or agencies who provide food to clients at no or very low cost, through food pantries, shelves, or hampers [8]. In Australia, food banks operate as both an ‘indirect’ and ‘direct’ service.

Food banks acquire food with funding from government, non-government, or philanthropic sources [9], but it is predominantly acquired through surplus, unsalable food redistribution at various points across the commercial food supply chain [10]. This includes food donations from agricultural production, manufacturing process defects (e.g. foods may be mislabelled, end of line, from damaged pallets or fail to meet cosmetic standards), wholesalers, supermarkets and the foodservice industry [11]. This food is subsequently transferred to food banks and other organisations for distribution.

In the absence of information regarding the number of clients serviced through the CFS, the universal measure of food bank performance is the total amount of food distributed (by weight) divided by a set amount to estimate the number of meals [12]. The Global FoodBanking Network estimated that 919 million kilograms (kg) of food was distributed in 2019, equated to 1.4 billion meals [13]. The United States (US) ‘Pounds per Person in Poverty’ (PPIP) metric assesses whether individuals receive enough food based on total pounds distributed to a specified area (county) by the total number of people in need [14]. With the primary focus being the redistribution of food waste, these measures provide insight into the amount and probable sufficiency of the food distributed (in terms of weight).

The current performance metric ignores key components essential for achieving food security, the regular acquisition of ‘…safe and nutritious food that meets dietary needs and food preferences…’ [15]. This is of concern given the increasing prevalence of food insecurity, the chronicity of use of food banks [7], and the high level of nutritional vulnerability of the client group [16]. As household incomes decline, healthy food purchases fall precipitously low [17] and the quality and variety of food is often compromised in order to satiate hunger [18]. As such, the dietary quality of food bank clients is poorer than that of the general population [19–22] and associated with nutrient inadequacy, metabolic changes, and subsequent development of diet-related chronic disease [23]. Obesity, diabetes and heart conditions are more prevalent in food bank clients than the general population [21].

A method to assess the dietary risk of donated food, which is aligned with the intention to address food security and meet clients’ needs is required. The term ‘dietary risk’ is typically used to describe population-based diet-related risk factors (e.g., a dietary pattern that does not meet public health recommendations for example, is low in fruit and vegetables or high in foods high in added sugar, salt or unsaturated fat) that contribute to the development of obesity and diet-related non-communicable diseases [24]. In the US, dietary risk criterion related to the dietary quality of an individual’s food intake are used in the certification of participants for government benefit programs (e.g., Special Supplemental Nutrition Program for Women, Infants and Children (WIC)) [25]. The concept of dietary risk is similar to that of food safety risk, and has previously been applied to the assessment of commercial food environments (e.g., supermarkets, restaurants) [26] to inform policy responses at a local level. Applying the concept of dietary risk to the food banking system and establishing criterion specific to donated foods (e.g., nutrition quality, food safety) will enhance assessments of the performance of the system in terms of the appropriateness of the food distributed to clients. Importantly, the intention is that the results can be used to initiate a quality improvement and assessment system (QIAS) for food policy. This is urgently needed given the marginalisation of food regulation and quality control in the CFS [27], and the documented food supply and organisational capacity constraints [28]. Assessing donated foods according to their dietary risk would demand more accountability from food donors and lead to food supply improvements across the CFS.

In recent decades, efforts have been made to improve the nutritional quality of donated foods in the CFS [29], however, the continued focus on the weight of food distributed by both food donors and food banks makes it difficult to prioritise the provision of healthier foods [30]. Research has shown that food banks are unable to provide their clients the amounts and types of food needed for a balanced diet [31]. There is an inadequacy in the supply of fruit and vegetables [20, 31], and dairy foods [31, 32] compared to national dietary recommendations.

Commercial food donations comprise 60% of food bank inventory in the US [33] and an estimated 80% in Australia [7]. The quality of the food provided by food banks is therefore dependent upon, and directly correlates with, food donations received [34]. Donors are primarily concerned with meeting their ‘zero waste’ commitments across their inventories, a major driver determining the types of products that are donated [35]. Donors, particularly supermarkets, publicise their efforts, and claim corporate social responsibility kudos for this redistribution of food waste [36].

The reliance on commercial food donations means that food banks have little autonomy over the types and amounts of food they distributed. The food distributed in the CFS continues to be criticised as being nutritionally poor [12] in the US [9, 37], United Kingdom (UK) [34, 38], Europe [20], Canada [39, 40] and Australia [16]. Clients themselves report that the food provided does not meet their cultural, social and health-related dietary needs [20, 41–45]. Provision of outdated and expired food is frequently reported [20, 41, 45, 46], and the consumption of these foods is unsafe and socially unacceptable [12]. Food safety must be emphasised given the vulnerability of both clients [47], and the system of delivery of surplus food [27]. Populations of lower socioeconomic status are suspected to experience greater rates of foodborne illness [48]. A Dutch study of food bank clients found almost all had received spoiled products on a regular basis [49]. Government regulations specifying that food must be donated within the set dates of consumption, with packaging integrity, and without signs of deterioration [50] appear to offer little protection to the client while donors are protected from any liability via Good Samaritan Laws [51, 52].

There are many examples of efforts to track and improve the nutritional quality of food bank inventories [53] such as ranking systems [54–56] and guidelines [57], which focus on making healthier choices easy and accessible to clients. Assessment and tracking of food safety is limited to evidence of implementation of food safety policy at food banks/pantries [58], or the exploration of staff knowledge and practices [59]. There are no initiatives that collectively measure dietary risk related to nutrition *and* food safety. There is a pressing need for a comprehensive approach to assessing the dietary risk of donated foods that is aligned with the definition of food security and meets the needs of clients accessing the CFS.

## Methods/Design

### Study aim

The aim of this audit protocol is to describe the method to assess the dietary risk of donated food, including the type, amount, nutrition quality, and safety of donated foods at Australian food banks. The protocol could also be applied to food banks in other countries or other organisations within the Australian CFS.

### Setting

The setting was a metropolitan branch of an Australian food banking organisation responsible for state-wide distribution of food (referred to as “the Food Bank” hereafter). The Food Bank operates out of a 6000sqm warehouse with six branches located throughout the state. This Food Bank selection was both convenient and purposeful as it is one of the largest charitable food relief providers in any Australian state. The nutrition quality of donated food has not been assessed previously in Australia using an audit approach that is objective, transparent, and rigorous, yet practical, acceptable and replicable is required.

### Study design

An audit of all food donated to and procured by the Food Bank was conducted over five consecutive days (May 2022). As the Food Bank is only opens on weekdays, this represents a week’s worth of food. The Standard Protocol Items: Recommendations for Intervention Trials (SPIRIT) checklist was used to guide this protocol.

### Audit process

#### Consultation and collaboration

Consultation with the Food Bank’s operational staff to organise the audit was undertaken from August 2021 to May 2022, including observing warehouse operations. A Memorandum of Understanding between the University and the Food Bank outlined the aims of research, data collection methods, and reporting activities.

#### Audit approaches

Different audit approaches were considered in an iterative process that spanned several months. A subset of Bowen et al. [60] areas of focus relating to ‘acceptability’ and ‘practicality’ was used to gauge the feasibility of different approaches. Acceptability relates to how those involved are predicted to react to the intervention [60]. Practicality is the extent, likelihood and manner in which an intervention can be delivered when resources, time, commitment or some combination thereof are constrained in some way [60]. The feasibility of interrogating delivery receipts, contacting donors for packing slips and auditing existing warehouse inventory, similar to the one-time inventory method described by Caspi [61] were considered. However, receipts lacked specific detail about the types of food donated and contacting donors was considered an unacceptable risk to donor/Food Bank relationships. A one-time stock inventory was considered impractical because of the requirement to use machinery (e.g., boom lift) to access warehouse shelves, and the time needed to be spent in cool storage and freezer areas. An audit of all incoming deliveries to the food bank warehouse over a set period was deemed the most feasible in terms of Food Bank staff burden (e.g., additional duties (acceptability)) and the researchers could work within the flow of the warehouse without disrupting usual operations (practicality). Although the number and nature of deliveries were unpredictable, the delivery window for each day was fixed between 0700 and 1500 when the warehouse was open for deliveries.

#### Food donations

All donated and procured food is sorted and distributed at the central Food Bank warehouse. The Food Bank receives food donations from growers, manufacturers, major supermarket chains, food service providers (e.g., institutional caterers), meal delivery companies and third-party logistics firms. Food donations are made for many reasons including: surplus stock resulting from over-ordering or under-selling, cancelled orders, changes in weather conditions or buyer preferences; production errors; damaged packaging; or because food is too close to the ‘Best Before’ date (BBD) [62]. The Food Bank permits donated foods that are close to their ‘Use By’ date (UBD) and up to six months past the BBD but food must not have any obvious signs of damage (e.g. broken packaging) or spoilage (e.g. mould). There are no nutrition guidelines or policies determining the types of food donated to the CFS in Australia. Similar to the UK, food banks in Australia are required to collect the donated food at a time and place convenient to donors, often with very little notice [63]. Community members also donate food directly to food banks.

#### Defining the food donation process

**Fig. 1:**
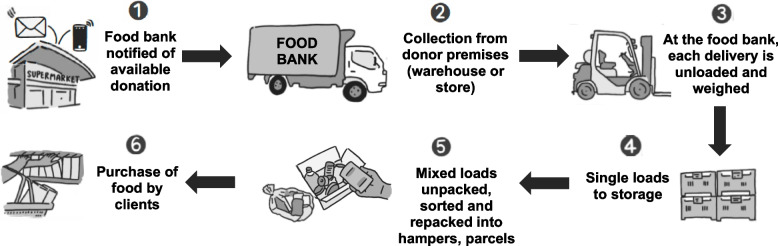
The Food Bank’s process for acquiring and sorting donated food

In consultation with the Food Bank’s operations staff, the process was defined in six stages, see Fig. 1. Each stage (steps 1 – 6) of the food donation/delivery process was defined to develop the audit procedures. Donations arrived as either single (same product) or mixed loads (unsorted, miscellaneous products).

#### Identifying audit attributes

This audit measured and documented the total weight (kilograms) of all deliveries received over the 5-day period using the total weight of each individual delivery, the total weight of each product within a delivery of mixed load, and the total weight by donor organisation. As the weight of food is the accepted performance measure for food banks, recording the weight of food audited, if repeated, directly contribute to reports of annual turnover.

Donor details and the following product information were collected including for packaged goods: brand and product name, product description and variety (if applicable); and, for fresh or unpackaged food: product description. Shelf-life category (frozen, chilled, or ambient) was also recorded.

The inherent quality of foods was categorized as either, ‘satisfactory’ or ‘damaged’ based on visual observations [27]. Food packaging protects products against contamination and quality loss [64]. Product packaging was categorised as damaged if the: packaging seal had been broken with/without the product exposed; packaging itself has been damaged (e.g., ripped or dented); or there was visible mould or the product was swollen/ ‘blown’ (an indication that the physical and microbial integrity has been compromised) [64]. Date marks were also recorded and provide a guide as to how long food can be kept before it begins to deteriorate, but still safe to eat (BBD) or becomes unsafe to eat (UBD) [65].

#### Summary of information audited

Table 1 details the audit attributes collected during the audit at the Food Bank warehouse:

**Table 1:** Summary of information audited

Data collected	Description
Digital images	Of each load and product
Date food was received	Date (day/month/year)
Donation or procurement details	Name of the donor or purchase program
Product information	Brand and product names Product description and variety If a whole/fresh/unpackaged food, a description of the product
Shelf-life category	Frozen, chilled or ambient
Weight	Total donated per load (kg) Number of products and pack sizes(g) Total weight of each product for mixed loads (kg)
Product quality	Damaged or satisfactory If damaged, a description
Date marking	Best Before and/or Use By date

For each audit attribute, corresponding research questions (Table 2) were formulated to inform both the audit process and data analysis stage.

**Table 2:** Audit attributes with corresponding to research questions and data collection requirements

Attribute	Research questions	Data required
Food received	• What is the total weight (kg) of food received (procured or donated)? • What proportion of food received was procured? • What proportion of food received was donated?	Weight of each delivery received Analysis of weight data
Donated food	• Who were the donor organisations? • What proportion of the total weight of donations is attributable to each donor? • What many types of products were donated? • How much variety existed across and within donations?	Name of donors Analysis using donor information and weight data Products assigned to 1 of 22 product categories and 1 of 163 product category groups
Nutritional quality	• How is donated food categorised within each group of AGTHE? • How is donated food categorised within each category of the NOVA system?	Brand and product names or whole food description, weight of each donation, total weight of donations received
Food safety	• What proportion of donated food was deemed safe to consume? • What proportion of donated food was deemed unsafe to consume? • What were the key reasons for unsafe foods? • Which donors are responsible for the donation of unsafe food?	Weight of each donation, total weight of donations received Product quality data Date marking data

#### Data collection process

A hand-held mobile device was used to take digital photographic images of all incoming deliveries. This method enabled quick data collection [66] within a busy warehouse setting. Food product images have been previously used to document nutritional quality of foods in the CFS [34, 38] and to monitor food environments more broadly [67]. Photographic images of each delivery were manually annotated electronically when taken [name of the donor; weight (kilograms); date marking (best before or use by date)]. Although manually annotating images on a mobile device is time consuming [68], within the busy warehouse setting, the single handheld device was an operationally effective way to capture deliveries. Other details [daily delivery number, donor, number of loads within delivery] were documented on paper to help keep track of all deliveries.

An audit framework (Table 3) was constructed using Microsoft Excel (Version 2019, Redmond, Washington, USA) profiling all donation and product attributes, enabling the methodical entry of each product photograph of each delivery once the audit was completed.

**Table 3:** Audit framework

Date (DD/MM/YY)																
**Delivery #**	**Donor/Purchase**	**Type of load**	**Product category**	**Product category group**	**Brand name**	**Product name**	**Product variety**	**Whole foods**	**Total amount donated (kg)**	**Pack size (g)**	**No. products/ pallets**	**Product quality**	**Product quality notes**	**Shelf-life category**	**Date marking**	
*(Free text)*	Donor/ Purchase *(Select)*	Single/Mixed *(Select)*	*e.g., “Frozen Food” (Select)*	*e.g., “Frozen fruit” (Select)*	As written on packaging *(Free text)*			Describe *(Free text)*	*(Free text)*	*(Free text)*	*(Free text)*	Satisfactory/ Unsatisfactory *(Select)*	Describe if unsatisfactory (*Free text)*	Ambient/Chilled/Frozen *(Select)*	UBD/BBD *(Select)*	Specify date *(Free text)*

#### Pilot test

The food donation audit process (Fig. 1) was reviewed with the Food Bank’s operations staff. Feedback led to the incorporation of the type and weight of the various delivery vessels (e.g., wooden pallets, cardboard produce bins, plastic ‘mega’ bins, metal cages) to the documentation. Researchers piloted the data collection tool on several incoming deliveries and the methodology was deemed appropriate for data collection because it was the most operationally effective way to capture delivery details. The process of data extraction was trialled using a sample of the pilot photos and the utility of the audit framework established.

#### Data collection

Two researchers (SM and MC) and two research assistants recorded deliveries on site in May 2022 for five consecutive days between 0700 to 1500. Data collection commenced at Step 3 (Fig. 1) and each delivery was sequentially weighed after being unloaded. Pre-determined weights of the delivery vessel were subtracted from the total weight. If donations arrived in a vessel where the weight was unknown, the delivery would be unpacked, then the vessel weighed separately.

One researcher took the images of the load and the other verbally confirmed the weight. Each image was then annotated [name of donor, total (gross) weight and date marking information]. Next, additional photos were taken of the product [brand and product names, variety, and pack/product size] and the total number of packs/products donated added. This was used to determine the total (net) weight of donations during the data analysis stage. Single product loads (deliveries of the same product) were typically transported to storage (Fig. 1 – Step 4). Mixed loads of unsorted, miscellaneous products were manually sorted so that each product could be counted (Fig. 1 – Step 5). Each unique product within each load was photographed to capture key product details.

Frozen mixed loads were unable to be sorted as the time required to sort, record and repack each product presented a food safety risk. Only details of the donor and total weight were recorded. Damaged products data collection required additional photographs to document the damage.

Warehouse staff were notified to dispose of items that were deemed unsafe for consumption, e.g., where packaging was ‘blown’, bloated or spoiled, leaking, crushed or obviously contaminated.

For quality control, researchers reviewed the photographs and notes at the end of each day to ensure every delivery had been accounted for. All photographs were date and time tagged by the device used. This enabled an assessment of efficiency of the audit methodology.

### Data extraction and analysis

A computer filing system was established with folders for each day of data collection and sub-folders for each delivery, organised chronologically by number and identified by donor name and product photographs were filed accordingly. The data on the annotated photographic images was extracted for each product and for each delivery according to categories in Table 3. Pre-coded responses were used to specify the type of load [single or mixed], product quality, shelf-life category and date marking type. Pre-coded product categories were assigned, [category (e.g., ‘Beverages’) and sub-category (e.g., ‘Carbonated Drinks’)].

Donor name, brand name, product name, product variety, whole food description, and product quality description and date marking all used free text. Quantities of each delivery was determined through the annotated photographic images, with the total (net) weight for single and mixed product loads calculated. The paper-based notes were used to confirm all delivery details.

Missing product information was obtained from the relevant food manufacturer website, visits to a local supermarket or contacting the food manufacturer directly, as sometimes important data was obscured on an image. For quality control, SM completed data entry, MC checked 10% of entries, and any discrepancies were discussed then reviewed by RG.

### Dietary risk assessment

#### Nutritional quality

The nutritional quality of audited products was assessed. Each product was categorised by two systems: 1) Food groups according to the Australian Guide to Healthy Eating (AGTHE) [69], and, the 2) level of food processing according to the NOVA classification [70]. The AGTHE, based on the Australian Dietary Guidelines (ADG) classifies food into the recommended five food groups (1) grain/cereal foods, 2) vegetables and legumes/beans, 3) fruit, 4) lean meat, poultry and alternatives, and 5) dairy and alternatives) and discretionary food (e.g. food and beverages high in fat, added sugar and/or salt) [69]. NOVA classification is according to the extent and purpose of processing (from unprocessed (Group 1), processed culinary ingredients (Group 2), processed (Group 3), to ultra-processed (Group 4)), with ultra-processed foods associated with dietary health risk [70]. The AGTHE is Australian Government’s food selection guide based on the recommendations of the ADGs. The AGTHE presents the serving size and proportions of food group foods recommended for daily consumption [69], and incorporates variety and nutrition adequacy recommendations [71]. Based on individual dietary recommendations, the types and proportions of food groups can be applied to food service, and in this case, assessment of ‘apparent consumption’ of the food supply. NOVA is a food-based classification system, informed by evidence of a food’s structure or composition, and associations with health outcomes [70]. Utilising both these systems considers the appropriateness of products in terms of both the role of food processing and dietary patterns on health outcomes [72, 73]. Nutrient-based classification systems were not used due to the reductionist focus on individual nutrients [71], however, the concept of nutrients ‘at-risk’ is incorporated into the development of the AGTHE. Importantly, it is the food supply, rather than single nutrients that are being assessed.

SM conducted the initial assessment, MC checked 10% of entries, with any identified discrepancies discussed, and reviewed by CEP.

#### Food safety assessment

Food safety is an important public health priority and the Australian Dietary Guideline #5 ‘Care for your food; prepare and store it safely’ [69] highlights the importance of food safety in terms of dietary risk. The proportion of damaged or unsafe products was determined as a proportion of the total number of entries rated as ‘unsatisfactory’ for product quality. Assessment was based on visual observations (the current practice in the CFS) [27]. Products deemed ‘unsatisfactory’ were categorized into 1) visibly damaged and rotting (e.g., food appears blown, swollen or has visible mould), 2) beyond date marking (past UBD or ≥ 6 months BBD), 3) broken or damaged packaging with product exposed, 4) broken or damaged packaging/label with product unexposed; or 5) other food safety concern (e.g., product recall).

### Data analysis

The audit comprised 1500 photographic images collected over 74 deliveries with only two deliveries procured over five days. Donors included supermarkets (n = 27), manufacturers (n = 11), the community (n = 9), growers (n = 8), logistics/distribution companies (n = 9), other food retailers (n = 3), meal delivery providers (n = 3), and inter-Food Bank branch donations (n = 2). The total weight of deliveries (procured and donated) was 108,509 kg, with donations accounting for 79% (86,050 kg) of the total weight of food received. Data analysis will be guided by the research questions in Table 2. All data will be entered into Microsoft Excel (Version 2019, Redmond, Washington, USA) and descriptive statistics such as frequencies applied.

Assessing these attributes against the criterion developed to answer the research questions will form the development of a tool to assess the dietary risk of donated food at Australian food banks, similar to FODR [26]. The tool will transcend the current weight-based performance indicator to increase the performance focus on a client-centred outcome by incorporating measures to assess dietary risk. The assessment of the apparent dietary quality and food safety of the donated food supply will better indicate performance against the definition of food security, and be used to initiate a Food Bank QIAS with transparent policy measures. The audit protocol was developed with quality improvement as the main intent, and this will be incorporated into development of the risk assessment criterion in the tool.

## Discussion

This protocol describes the methods developed to identify and assess the dietary risk of donated food at an Australian food bank, incorporating the quantity *and* quality of food received. The requirement for this protocol is based on the influential role that food banks play in shaping the food environment of their food insecure clients [74]. The audit process could not have been developed and implemented without the express permission and willingness from the Food Bank leadership, management and warehouse staff. The regular visits, respectful communication and collaboration with staff at all stages, but particularly the planning phase was a high priority to ensure organisational ‘buy-in’ [75].

During the audit, researchers worked within the ‘flow’ of the warehouse, being mindful to remain unobtrusive and allow staff to undertake operations as normal. The use of a hand-held mobile device to take photographic images of all incoming deliveries proved to be quick, effective, and an unobtrusive, therefore an efficient and objective way to collect audit data in a busy warehouse setting. This protocol could be utilised to assess donations in other food bank locations in Australia, or other CFS settings, such as food rescue organisations. Preliminary audit results revealed the donation of over 85,000 kg of food during the five consecutive days of data collection and provides an indication of the scale of the food bank operations [5]. Absolute and relative weights (total kilograms and percentage of total kilograms) of the audit data for each food category and each subcategory provide important, previously unavailable information, when considering overall dietary risk of the CFS. Understanding the nature of the food supply in terms of the type, amount, safety, and nutritional quality and source (donor) of food donated provides food banks with information about each donors’ role in supporting their food insecure clients and CFS partners. The dietary risk assessment of the food provided by each donor can assist evidence-based discussions about how they can assist in providing an appropriate and acceptable food supply for the CFS and to initiate conversations for feasible and acceptable actions to improve the nutrition quality and food safety of donated food.

Donors will likely require an explanation of the rationale behind key decisions such as rejecting food donations [76]. Profiling the nutrition quality of donated food provides timely and tangible information to underpin discussions, important given the vulnerability of food bank clients [47], who are at an exacerbated risk of diet-related chronic disease [77] and increasingly experiencing long term food deprivation [78]. The food received from the CFS can represent over half of a clients’ total daily dietary intake [32] and consistent evidence shows that clients would prefer healthy foods including meat, dairy products, fresh fruit, and vegetables [79–85]. In the Netherlands, clients who received food parcels containing an abundance of products high in fat and sugar said they felt like they weren’t being taken seriously as adults with responsibilities for providing healthy meals [49]. The nutrition assessment results will establish a baseline of the nutrition quality of donated food in Australia, and their contribution to dietary risk. Without this data, food banks are unable to effectively assess the healthfulness of the food they distribute or measure the progress they make toward improving nutritional quality [35].

Measuring donations of damaged and unsafe food is important given the global lack of data on food safety in the CFS [27] and existing evidence that lower socioeconomic groups are suspected to experience greater rates of foodborne illness [48]. Further, clients consider the provision of unsafe food an erosion of their dignity: “*Should we get food poisoning because this food is left over, and I am poor?*” [49]. Clients expressed dissatisfaction at receiving foods ‘close to’ or past the expiry date [86] in one Australian study, and, in another, they highlighted concerns for their own safety around ‘out of date’ foods, which are ‘scary’ [46]. These examples highlight the integration gap between food safety controls in the conventional food supply chain and that of the CFS [27], and the need for an audit such as this to quantify the presence of foods that pose a dietary health risk to clients.

The CFS is no longer likely to meet the nutritional and social needs of its clients who are experiencing food insecurity [87], due to increasing demand for food and the inappropriateness of the donated food supply. Despite this, the system continues to contribute to corporate welfare [52]. Commercial donors, particularly supermarkets, win big: they get to donate outdated, expired, and unsaleable food, receive tax write-offs and are protected from liability [88]. Further, supermarkets claim corporate social responsibility kudos by framing partnerships with the CFS as being part of the solution to food waste [36]. Indeed, surplus food redistribution is hailed as a ‘win–win’ strategy to address household food insecurity and food waste [89]. But the current system of food redistribution represents a public health risk to an already vulnerable client group and this research acknowledges the need for more transparency and accountability from food donors. There are potential benefits for donors as they are better able to report against their corporate social responsibility statements given that the donation of surplus food and the reduction of food waste are key focus areas for demonstrating good corporate citizenship [90].

Audit results can be used to initiate a QIAS. Establishing QIAS within food banks can improve core operational processes, and reduce costs associated with poor quality and/or damaged food, as well as to inform and guide collective actions to improve the CFS food supply, pushing past the old paradigm, which emphasised *any* kind of food, toward a focus on sufficient, safe and nutritious food.

### Strengths and limitations

A key strength of this research is that the data collected has previously been inaccessible and will be used to assess the quality and dietary risk of donated food at an Australian food bank. This will be an important contribution to the literature as there is increasing interest in the effectiveness, efficiency and equity of food banks [14, 28]. The process of developing the audit has already enabled the sharing of an understanding of the issues facing CFS operations in Australia, and the finding will deepen this understanding. This protocol could be replicated or adapted for use in other food banks, indirect or direct service CFS organisations, such as food rescue organisations.

Another strength of the research is that the protocol was undertaken with the full support and engagement of the Food Bank, following extensive collaboration. A unique part of this research is positioning and composition of the research team, all five researchers are experienced public health nutritionists, and four have close relationships with the Food Bank. At the time of the research, RG was the Food and Nutrition Manager, MC was a staff member, SM was a volunteer and PhD candidate, and CMP was a Board Member, and CEP is a public servant working in a health service. Although this may be perceived as a potential conflict of interest, the composition of the team enabled the study and strengthened the collaboration. The positionality of the research team and engagement of Food Bank staff enables an integrated, cross disciplinary approach [91]. This will assist with co-creation of ongoing actions and means that the audit process is more likely to be embedded into the Food Bank’s operations.

A limitation of the research is that although the hand-held mobile device with manual annotations proved effective, the pace of the warehouse activity and the sheer number of deliveries meant that some details on single products audit were missed. This potential limitation was overcome by collection of missing data from other sources, such as supermarket websites or instore visits, during data extraction. Future audits at the Food Bank or at other food bank branches would provide additional data and enable the analysis of similarities and differences of the dietary risk of donated food in Australia.

## Conclusion

There is an urgent need to establish the dietary risk profile of donated food in Australian CFS. Food banks play an influential role in shaping the food environment of individuals experiencing food insecurity [74]. The provision of nutritionally poor, socially inappropriate and devalued food [92] presents a dietary risk to a client group who are already rendered susceptible to increased health risk [93] due to social determinants, these people are increasingly reliant on food banks [34]. Determining the dietary risk of donated food can help initiate and inform constructive discussion with donors, to contribute to improvements across the sector to better meet the needs of clients experiencing severe food insecurity.

## Data Availability

The database variables compiled during the current study are available from the corresponding author upon reasonable request.
